# Supplementary material for: A Doxorubicin-Glucuronide Prodrug Released from Nanogels Activated by High-Intensity Focused Ultrasound Liberated β-Glucuronidase
Source: Pharmaceutics. 2020 Jun 10;12(6):536. doi: 10.3390/pharmaceutics12060536 (PMC7355552; doi:10.3390/pharmaceutics12060536)
Supplement: Supplementary file 1 [file pharmaceutics-12-00536-s001.pdf]

pharmaceutics

MDPI

Supplementary Materials: A Doxorubicin-Glucuronide Prodrug Released from Nanogels Activated by High-Intensity Focused Ultrasound Liberated β-Glucuronidase

Helena C. Besse, Yinan Chen, Hans W. Scheeren, Josbert M. Metselaar, Twan Lammers, Chrit T.W. Moonen, Wim E. Hennink and Roel Deckers *

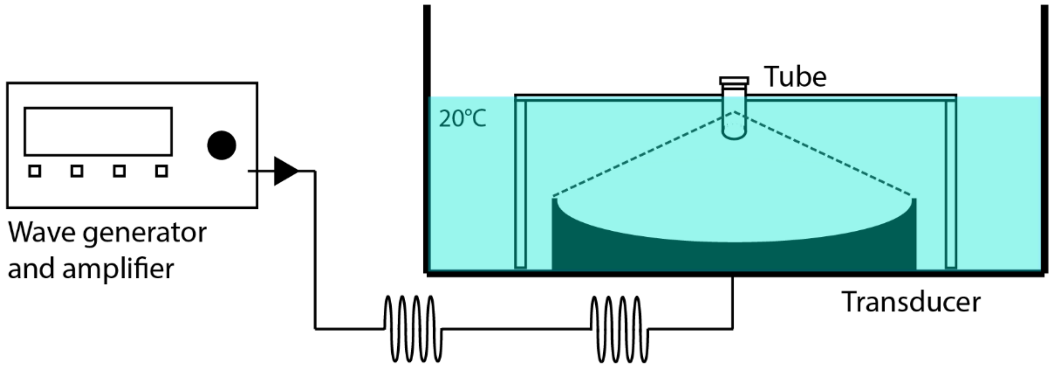

Figure S1. Schematic representation of the in-house-build HIFU setup, consisting of a transducer, an amplifier, an oscilloscope, a wave generator, a hydrophone, and a sample holder. HIFU was performed by a single element focused ultrasound transducer (Imasonic, Besançon, France). During HIFU treatment, a PCR tube (Bio rad, California, USA), containing the sample, was positioned in the sample holder in the focus of the ultrasound beam.

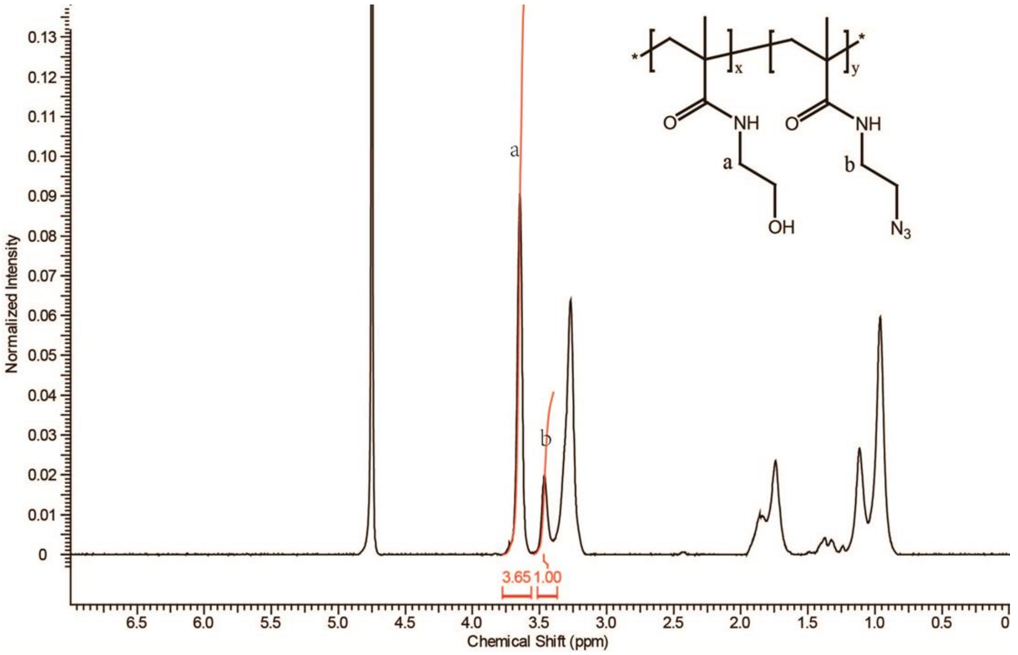

Figure S2. ^{1}H-NMR spectrum of p(HEAm-co-AzEMAm), from [1], reprinted with permission from Royal Society of Chemistry, 2020.

Table S1. Characteristics of p(HEMAm-co-AzEMAm) as determined by ^{1}H-NMR, UPLC and GPC, obtained from [1], reprinted with permission from Royal Society of Chemistry, 2020.

HEMAm/AzEMAm mol/mol in the feed	Yield [%]	Copolymer composition (by 1H-NMR)	Conversion [%] (by UPLC)		M_a [kDa] (by GPC)	PDI (by GPC)
			HEMAm	AzEMAm		
**80/20**	95.6	79/21	98.8	99.1	14.6	3.0

References

1 Chen, Y.; Tezcan, O.; Li, D.; Beztsinna, N.; Lou, B.; Etrych, T.; Ulbrich, K.; Metselaar, J.M.; Lammers, T.; Hennink, W.E. Overcoming multidrug resistance using folate receptor-targeted and pH-responsive polymeric nanogels containing covalently entrapped doxorubicin. *Nanoscale* **2017**, *9*, 10404–10419, doi:10.1039/c7nr03592f.
